# Difference in outcomes between right and left posterior descending artery percutaneous coronary intervention: an Australian statewide registry analysis

**DOI:** 10.1186/s43044-026-00755-2

**Published:** 2026-06-08

**Authors:** Muhtasim Rahman Zahin, Riley Batchelor, Angela Brennan, Diem Dinh, Christopher Reid, Dion Stub, Jeffrey Lefkovits

**Affiliations:** 1 Royal Melbourne Hospital, Melbourne, Australia; 2 Monash University, Melbourne, Australia; 3 Curtin University, Perth, Australia

## Abstract

**Introduction:**

Percutaneous coronary intervention (PCI) is an important treatment for coronary artery disease, and coronary dominance may influence procedural risk. PCI to a left-sided posterior descending artery (L-PDA) occurs in the setting of left-dominant coronary anatomy and may involve greater anatomical complexity and a larger myocardial territory at risk than PCI to a right-sided PDA (R-PDA). We performed a retrospective analysis of a statewide registry to compare the characteristics and outcomes of R-PDA and L-PDA PCI.

**Methods:**

The Victorian Cardiac Outcomes Registry is a state-wide quality registry with all PCI capable centres in Victoria, Australia contributing. We undertook a retrospective analysis of patients undergoing PCI for L-PDA and R-PDA lesions between 2013 and 2022.

**Results:**

2,880 patients were included over the 10-year study period, with 2,282 (79.2%) undergoing R-PDA PCI and 598 (20.8%) undergoing L-PDA PCI. Patient characteristics between groups were of similar age (66.3 years in both groups), gender (17.9% vs. 14.5%; *p* = 0.06) and had comparable rates of comorbidities including diabetes (25.8% vs. 24.9%; *p* = 0.66). There was no significant difference between groups with respect to 30-day mortality (1.1% vs. 0.5%; *p* = 0.21). 30-day MACE was also comparable (2.2% both groups; *p* = 0.93).

**Conclusions:**

Data from a large contemporary cohort did not find any association between coronary dominance and clinical outcomes when undertaking PDA PCI.

## Introduction

Percutaneous coronary intervention (PCI) is a cornerstone of treatment for coronary artery disease, but procedural risk varies according to both patient and lesion characteristics [1–3]. Coronary artery dominance, defined by the anatomical origin of the posterior descending artery (PDA), has previously been identified as a potential determinant of outcome following PCI [4–6]. In most patients, the PDA arises from the right coronary artery (right-dominant circulation), whereas in a smaller proportion it arises from the left circumflex artery (left-dominant circulation) or from both systems [2]. Prior studies have suggested that left coronary dominance may be associated with worse outcomes after PCI. Proposed explanations include delayed recognition of left circumflex-related ischaemia, greater angiographic complexity, and differences in myocardial territory at risk, with right-dominant anatomy potentially providing more redundant perfusion through the right coronary artery. However, these studies have largely evaluated unselected acute coronary syndrome populations and have not accounted for culprit lesion location. We therefore specifically examined PCI to the PDA, which generally subtends a relatively small myocardial territory, to determine whether the previously reported association between coronary dominance and adverse outcomes persists when the culprit lesion is confined to the PDA.

## Methods

The Victorian Cardiac Outcomes Registry (VCOR) is a statewide quality registry incorporating data from all PCI-capable centres in Victoria. It collects PCI data including 30-day mortality, 30-day unplanned revascularisation and 30-day major adverse cardiac and cerebrovascular events [1]. We performed a retrospective analysis of patients undergoing PCI for posterior descending artery (PDA) lesions between 2013 and 2022. Patients were classified according to the origin of the PDA as either right-sided (R-PDA), arising from the right coronary artery, or left-sided (L-PDA), arising from the left circumflex artery. Patients with balanced coronary circulation were included and categorised according to the artery used to treat the PDA lesion. These cases represented a minority of the cohort. Clinical outcomes of interest included in-hospital mortality, major bleeding, stent thrombosis, 30-day mortality, and 30-day major adverse cardiovascular events (MACE). Continuous variables are presented as mean ± standard deviation and were compared between groups using the Student t-test or Mann–Whitney U test, as appropriate. Categorical variables are reported as counts and percentages and were compared using the chi-squared test or Fisher exact test, as appropriate.

## Results

A total of 2,880 patients were included, of whom 2,282 (79.2%) underwent R-PDA PCI and 598 (20.8%) underwent L-PDA PCI. Baseline characteristics were broadly similar between groups, with no significant differences in age, sex, or comorbidities including diabetes, prior stroke, peripheral vascular disease, and previous coronary artery bypass grafting. The indication for PCI was also comparable, with 59.7% of all procedures performed for non-ACS presentations. Femoral access was used more frequently in the R-PDA group than in the L-PDA group (35.6% vs. 29.1%, *p* = 0.003), whereas other procedural characteristics, including device type and total stent length, were similar between groups (Table 1).

**Table 1:** Baseline data for R-PDA and L-PDA PCI patients

Factor		Total	*R*-PDA	L-PDA	*p*-value
N		2880	2282	598	
Age, mean (SD)		66.3 (11.4)	66.3 (11.5)	66.3 (11.2)	0.88
Gender	Male	2385 (82.8%)	1874 (82.1%)	511 (85.5%)	0.055
	Female	495 (17.2%)	408 (17.9%)	87 (14.5%)	
Length of Stay, median (IQR)		2.0 (1.0, 4.0)	3.0 (4.9)	2.8 (3.0)	0.28
Body mass index, median (IQR)		28.1 (25.2, 31.7)	28.1 (25.3, 31.7)	27.9 (25.1, 31.7)	0.74
Diabetes history		738 (25.6%)	589 (25.8%)	149 (24.9%)	0.66
Peripheral vascular disease		80 (2.8%)	65 (2.8%)	15 (2.5%)	0.65
Cerebrovascular disease history		85 (3.0%)	73 (3.2%)	12 (2.0%)	0.13
Previous PCI		1363 (47.3%)	1067 (46.8%)	296 (49.5%)	0.23
Previous CABG		245 (8.5%)	202 (8.9%)	43 (7.2%)	0.19
eGFR	missing	183 (6.4%)	127 (5.6%)	56 (9.4%)	< 0.001
	egfr > 60	2196 (76.3%)	1730 (75.8%)	466 (77.9%)	
	egfr 30–60	448 (15.6%)	381 (16.7%)	67 (11.2%)	
	egfr < = 30	53 (1.8%)	44 (1.9%)	9 (1.5%)	
Type of ACS	STEMI	414 (14.4%)	337 (14.8%)	77 (12.9%)	0.65
	NSTEMI	540 (18.8%)	422 (18.5%)	118 (19.7%)	
	UAP	207 (7.2%)	165 (7.2%)	42 (7.0%)	
	Non-ACS	1719 (59.7%)	1358 (59.5%)	361 (60.4%)	
Ejection Fraction	missing	352 (12.2%)	268 (11.7%)	84 (14.0%)	0.64
	Normal	1908 (66.3%)	1519 (66.6%)	389 (65.1%)	
	Mild	387 (13.4%)	308 (13.5%)	79 (13.2%)	
	Moderate	162 (5.6%)	131 (5.7%)	31 (5.2%)	
	Severe	71 (2.5%)	56 (2.5%)	15 (2.5%)	
Angiographic characteristics					
Femoral access		987 (34.3%)	813 (35.6%)	174 (29.1%)	0.003
Lesion type (ACC/AHA classification)	A	204 (7.1%)	164 (7.2%)	40 (6.7%)	0.021
	B1	954 (33.1%)	730 (32.0%)	224 (37.5%)	
	B2	1026 (35.6%)	812 (35.6%)	214 (35.8%)	
	C	696 (24.2%)	576 (25.2%)	120 (20.1%)	
Device type	Any DES	2615 (90.8%)	2056 (90.1%)	559 (93.5%)	0.18
	Any BVS no DES	2 (0.1%)	2 (0.1%)	0 (0.0%)	
	BMS only	67 (2.3%)	59 (2.6%)	8 (1.3%)	
	other stent	1 (< 1%)	1 (< 1%)	0 (0.0%)	
	POBA only	138 (4.8%)	115 (5.0%)	23 (3.8%)	
	No stent/balloon	57 (2.0%)	49 (2.1%)	8 (1.3%)	
Stent length (mm)		26.0 (16.0, 43.0)	26.0 (16.0, 44.0)	24.0 (16.0, 41.0)	0.46

*eGFR* estimated glomerular filtration rate,* ACS* Acute coronary syndrome,* STEMI* ST segment elevation myocardial infarction,* NSTEMI* Non ST segment elevation myocardial infarction,* UAP* Unstable angina,* DES* Drug eluting stent,* BVS* Bioresorbable vascular scaffold, BMS: Bare metal stent,* POBA* Plain old balloon angioplasty

The primary outcome of in-hospital mortality was low overall and did not differ significantly between groups, occurring in 18 patients (0.8%) in the R-PDA group and 2 patients (0.3%) in the L-PDA group (*p* = 0.23); similarly, 30-day mortality was 1.1% versus 0.5%, respectively (*p* = 0.21). Rates of other clinical outcomes, including 30-day MACE and target vessel revascularisation, were also low and comparable between groups, with no statistically significant differences observed.

## Discussion

Our retrospective analysis included 2,880 patients undergoing PCI to the PDA and specifically examined the association between coronary dominance and outcomes in this lesion subset. Consistent with established anatomical distributions, 79% of patients had right-dominant circulation and 21% had left-dominant circulation. Femoral access was used in 34.3% of patients in our cohort. This should be interpreted in the context of the 10-year study period from 2013 to 2022 during which time access-site practice changed and radial access became increasingly prevalent [2]. The principal finding was that clinical event rates were low overall, with no statistically significant differences in in-hospital mortality, 30-day mortality, 30-day major adverse cardiovascular events, or target vessel revascularisation between the right- and left-dominant groups.

The published literature examining coronary dominance and PCI outcomes remains limited and has largely suggested less favourable outcomes in patients with left-dominant coronary anatomy. Kuno et al. reported higher in-hospital mortality among left-dominant patients undergoing PCI [3]. In the TWENTE trial cohort, Lam et al. found that left dominance was independently associated with periprocedural infarction and recurrent myocardial infarction over 2 years, although mortality did not differ significantly [4]. Parikh et al. also reported higher in-hospital mortality after PCI for ACS in patients with left-dominant circulation, particularly when the culprit territory involved the left main or left circumflex artery [5]. Among STEMI cohorts, Veltman et al. observed higher 30-day mortality and reinfarction in left-dominant patients, although long-term outcomes were similar [6], whereas Mikaeilvand et al. found no mortality difference but reported poorer surrogate markers of reperfusion and ventricular function in the left-dominant group [7]. A meta-analysis by Khan et al. similarly suggested an association between left coronary dominance and increased mortality after PCI [8].

Several mechanisms have been proposed to explain these findings. These include delayed recognition of left circumflex-related ischaemia on standard 12-lead ECG, a larger myocardial territory at risk in left-dominant systems, and the possibility that a right-dominant circulation provides greater perfusion redundancy through the right coronary artery and its branches [3, 9]. Prior work focused on left circumflex occlusion has also suggested that right-dominant anatomy may attenuate the clinical severity of ischaemia, with more frequent NSTEMI rather than STEMI presentations in some cohorts [9]. In addition, technical factors may be relevant, as a dominant left circumflex artery may be associated with greater angiographic complexity and less favourable procedural geometry in selected cases [10].

However, an important distinction between our study and most prior reports is the anatomical focus of the culprit lesion. Previous studies have generally assessed broad ACS or PCI populations without isolating lesion location and therefore may have been heavily influenced by proximal culprit lesions involving the left main or proximal left circumflex arteries, where the jeopardised myocardial territory is substantially larger. By contrast, the present study specifically examined PCI to the PDA. When the culprit lesion is confined to the PDA, the amount of myocardium at risk is likely smaller than in more proximal left-sided lesions, which may reduce any effect of coronary dominance on short-term clinical outcomes. Our findings therefore suggest that the adverse prognostic signal previously attributed to left dominance may not be uniform across all lesion locations and may instead be more relevant in lesions subtending larger myocardial territories.

These findings should be interpreted in the context of several limitations. This was a retrospective observational analysis and is therefore subject to selection bias, unmeasured confounding, and the inherent limitations of registry-based data. No matched or propensity-adjusted analysis was performed, which limits inference. We also did not assess infarct size, collateralisation, lesion complexity, or procedural characteristics in sufficient detail to determine mechanistic explanations for the observed findings. The imbalance in group size reflects the underlying prevalence of coronary dominance patterns but may also have reduced power to detect small differences in uncommon clinical events. In addition, follow-up was limited to 30 days in accordance with the registry data collection protocol, precluding assessment of longer-term prognosis, and the registry reflects Australian practice, which may limit generalisability to other healthcare settings. Finally, patients with balanced coronary circulation represented a minority of cases and were not analysed as a separate subgroup, which may limit interpretation in this anatomical subset (Table 2).

**Table 2:** Table demonstrating clinical outcomes between R-PDA and L-PDA lesions

Factor	All	*R*-PDA	L-PDA	*p*-value
N	2880	2282 (79%)	598 (21%)	
In hospital mortality	20 (0.7%)	18 (0.8%)	2 (0.3%)	0.23
Major bleed	14 (0.5%)	14 (0.6%)	0 (0.0%)	0.055
Stent thrombosis	3 (0.1%)	3 (0.1%)	0 (0.0%)	0.38
30-day mortality	27 (0.9%)	24 (1.1%)	3 (0.5%)	0.21
30-day MACE	64 (2.2%)	51 (2.2%)	13 (2.2%)	0.93
Target vessel revascularisation	8 (0.3%)	6 (0.3%)	2 (0.3%)	0.77

*MACE* Major adverse cardiovascular events

In conclusion, among patients undergoing PCI to the PDA, left-dominant coronary anatomy was not associated with worse short-term clinical outcomes compared with right-dominant anatomy. These findings differ from prior studies of broader PCI and ACS populations and suggest that the relationship between coronary dominance and prognosis may be modified by culprit lesion location. Further studies with detailed anatomical and procedural data, and with longer-term follow-up, are needed to clarify the prognostic relevance of coronary dominance across different coronary territories.

## Data Availability

No datasets were generated or analysed during the current study.

## Abbreviations

PDA: Posterior descending artery
R-PDA: Right posterior descending artery
L-PDA: Left posterior descending artery
ACS: Acute coronary syndrome
PCI: Percutaneous coronary intervention
